# Improved data retrieval from TreeBASE via taxonomic and linguistic data enrichment

**DOI:** 10.1186/1471-2148-9-93

**Published:** 2009-05-08

**Authors:** Nadia Anwar, Ela Hunt

**Affiliations:** 1 Faculty of Biomedical and Life Sciences, University of Glasgow, UK; 2 Computer and Information Sciences, University of Strathclyde, UK

## Abstract

**Background:**

TreeBASE, the only data repository for phylogenetic studies, is not being used effectively since it does not meet the taxonomic data retrieval requirements of the systematics community. We show, through an examination of the queries performed on TreeBASE, that data retrieval using taxon names is unsatisfactory.

**Results:**

We report on a new wrapper supporting taxon queries on TreeBASE by utilising a Taxonomy and Classification Database (TCl-Db) we created. TCl-Db holds merged and consolidated taxonomic names from multiple data sources and can be used to translate hierarchical, vernacular and synonym queries into specific query terms in TreeBASE. The query expansion supported by TCl-Db shows very significant information retrieval quality improvement. The wrapper can be accessed at the URL

The methodology we developed is scalable and can be applied to new data, as those become available in the future.

**Conclusion:**

Significantly improved data retrieval quality is shown for all queries, and additional flexibility is achieved via user-driven taxonomy selection.

## Background

Systematics aims to increase our understanding of biological diversity by identifying and classifying organisms and using phylogenies to understand the relationships between organisms. The field has developed very elaborate and sophisticated tools for phylogeny construction, and practitioners have been very active in building new, better and faster algorithms [1,2]. However, this has not been matched with database development for long term access and storage of the phylogenies produced by these algorithms. Although much of the data used in phylogenetic analysis is acquired from databases in other fields, particularly specimen data from museum collections [3] and sequence data [2] such as those available at NCBI [4], the results of phylogenetic analysis are not as easily accessible. Mostly, phylogenetic data are retrieved through literature searches and remain buried in the pages and supplementary material sections of the journals in which they are published. This inaccessibility of data compounds the practicality of its use and limits the full potential of information reuse. Projects such as the Tree of Life [5] face significant data accessibility issues.

The Tree of Life aims to build a complete phylogenetic tree of the world's biodiversity, and to ultimately describe the history of life on earth. The informatics requirements are considerable, as the available data collections grow in size and complexity. Confronting the information explosion requires creative new approaches to facilitating the use of that information. Finding information in complex data sets becomes increasingly difficult as the data grow, therefore data search and discovery needs to be timely, intuitive and precise. Data retrieval through meaningful queries [6] is paramount to the successful fulfilment of the ever more sophisticated data requirements of the systematics community. A phylogenetic data repository [7] should have a good understanding of the organisms that are represented in the phylogenetic trees and support searches using species and higher taxa names. However, currently this is not the case. TreeBASE [8] is currently the only repository for phylogenetic analyses. Here we show that data retrieval using taxonomic names as query terms is inadequate.

In the GenBank  sequence data base, which contains the NCBI taxonomy, a query can be performed to retrieve all insect sequences or all *Drosophila *sequences. TreeBASE, however, does not contain a taxonomy and queries selecting all *Drosophila *studies or phylogenetic trees for insects are not easily specified. The inclusion of a taxonomic infrastructure within TreeBASE is essential to support such queries.

To address the problem of TreeBASE querying, we designed a taxonomic data warehouse combining taxonomic names and classification data that can be *superimposed *on TreeBASE to enable hierarchical and linguistic query expansion. Our hypothesis was that data integration in a warehouse would also provide breadth of coverage for taxon names by combining data from multiple sources.

The rest of this paper is structured as follows. The next section provides background on taxonomy and its uses in systematics. An outline of the user requirements and a description of TCl-Db, the data warehouse built as a taxonomic infrastructure for TreeBASE, and the methods of query expansion are then given. Finally, we show retrieval problems experienced by TreeBASE users through an analysis of the query logs from TreeBASE. We conclude that data retrieval difficulties are in part due to the lack of taxonomic intelligence in TreeBASE, and we demonstrate improved data retrieval based on the use of TCl-Db and the software infrastructure we created, as compared to results delivered by Phylofinder [9].

### Taxonomy

Taxonomic data are produced by the processes of *Naming*, which involves attaching a label to a concept for the purposes of communication, and *Classification*, that is arranging similar concepts together for the purpose of organisation. The name provides a handle on the biological organism and the position in the classification provides knowledge of the organism in terms of its similarity to others [10]. This section gives a brief overview of the difficulties users experience when utilising taxonomic data.

The taxonomic classification system is an information storage and retrieval system [11], originally designed to be easily memorised [12]. Taxon names serve two roles; the name represents an organism that was described and named by a taxonomist and the name is also placed in a hierarchy to relate the organism to the tree of life. This duality presents difficulties in the use of taxonomic names. The interdependence between the name and the classification, the fact that names are not necessarily unique to one organism and also that the placement of an organism's name into the hierarchy is not fixed, all complicate the use of taxonomic names for information storage and retrieval. Compounding this is the distributed nature of the data. The taxonomy field uses over 200 information systems . This number will continue to grow as herbariums and museums digitise their collections [13] and make their data accessible on the web. Although taxonomy has firmly taken its place as a digital science, data accessibility continues to cause difficulty; with the distribution there is also the heterogeneity of the data and the lack of one all encompassing taxonomic reference. Given that the amount of data is growing and the data is in constant flux, it is unlikely that it will be possible to agree on a 'unitary taxonomy' [14]. However, a single all encompassing data portal is achievable [15], and this challenge is being addressed by GBIF [16] and projects such as the Encyclopaedia of Life [17].

Most taxonomic data systems were developed to meet particular requirements in their use or data scope. Taxonomic data is, by its nature, distributed. The data produced from taxonomic research tends to follow a particular focus, a group such as insects or birds, or a geographical location, or a period in history. There is significant heterogeneity in the data models and storage formats of the databases and the interfaces provided to access the data. The taxonomic community have established the Taxonomic Databases Working Group (TDWG) to address data standards, data integration and interoperability. This effort is beginning to alleviate some of the accessibility and interoperability problems experienced by users [18]. Taxonomic data are also not easily deployed outside the systems in which they are stored. This is due to the nature of taxonomic names. As stated in [19], taxa are not facts like the data in most other databases, instead, taxa are hypotheses which are "proposed, used, modified, and then perhaps discarded, as evidence dictates". The classification of an organism is based on a set of criteria selected by the expert taxonomist. Not only do these criteria change, for example, sequence versus morphology, [20], but also different criteria are used by different taxonomists (different morphological characteristics can be given different weights).

Additional complications arise from the addition of new data as new organisms are discovered, and taxonomic revisions that are made to update existing groups. There can be, at any one time, more than one accepted taxonomic opinion on the name and classification of an organism. This complicates the use of taxon names as search terms, as the meaning of the names can change. For example, in situations where a name has changed for taxonomic reasons, such as *Diomedea albatrus *which was changed to *Phoebastria albatrus *[21], additional support is needed to recognise that relevant data may be attached to both of these terms. When the user performs a search on *Phoebastria albatrus*, should any data associated with *Diomedea albatrus *also be returned? Similarly, when a user performs a search on a vernacular term 'short-tailed albatross', is it assumed that the system should translate this term to the appropriate Latin names, i.e. *Phoebastria albatrus *and *Diomedea albatrus*? Also, when a search is performed on the term Aves, we need to know whether the user requires the NCBI meaning of the term or the ITIS [22] meaning of the term. It is not surprising that at the time of development the TreeBASE developers shelved these taxonomic issues. It is now timely and important to address the taxonomic requirements of TreeBASE, given that the system is in the process of being overhauled by the CIPRES project [23].

CIPRES, CyberInfrastructure for Phylogenetic RESearch have taken over responsibility for TreeBASE and as part of their database research programme, they plan to overhaul the database to enable more complex queries than those currently available in TreeBASE. The new version of TreeBASE is named TreeBASE2 and the published Entity-Relationship model contains a taxon module from which it appears that the taxonomic data will be curated from external data sources. However, the documentation does not suggest that hierarchical queries will be directly supported by the TreeBASE2 schema. In addition to TreeBASE2, the CIPRES project have two other research programmes: algorithms for phylogenetic reconstruction and visualisations; and a modelling programme that aims to build mathematical models that can be used to test phylogenetic reconstructions. The project aims to build a complete infrastructure of data and algorithms for the systematics community.

### Systematics

Like taxonomists, most systematists focus their research on a particular group. For these scientists the taxonomic requirements are fairly manageable, and usually involve the most up-to-date checklists. Most scientists are adept at keeping up-to-date with the literature in their area and for the most part they produce their own data. Some systematics studies, however, go beyond the usual boundaries of collecting data and building trees. Two examples are cospeciation analysis [24] and the study of species richness [25]. A cospeciation study usually follows two taxonomic schemes: one for the host species, and one for the parasites. Parasites are of particular interest in systematics because of the shared history of the host and the parasite [24,26]. The analysis involves comparing the phylogenies of the parasite and the host. These phylogenies either need to be collected from the literature or built from morphological or sequence data. For the data that are collected, literature searches are normally conducted using the species or higher taxa names as the search terms. Similarly, a study of the parasite species richness of a group of organisms also uses two taxonomic schemes and involves collecting data using taxon names as search terms [27]. These examples exemplify that more studies now require gathering, not just previously published data in order to stay up-to-date, but also, data collection for further analysis. Another example, where collecting data is integral to the study, is in building super trees [28,29].

Within super tree analyses, data from several studies are gathered using taxon names as search terms. Once these data are collected, the taxonomic names across these data need to be synonymised. Usually, this is done through one authoritative source, for example, Beck *et. al*. [30] used Mammal Species of the World [31]; and Thomas *et. al*. [32] used the taxonomy of Sibley and Monroe [33]. Where one such data source exists, this is a simple task, however, the time is approaching when super trees go beyond the use of one taxonomic source [5].

The main use of taxonomic data outside its immediate user community is in information retrieval, as the examples above show. Names are used as the keys to retrieve data [34-36]. Currently, no one taxonomic data provider supports the needs of the systematics community. Despite TreeBASE being the only repository for phylogenetic data, systematists prefer to gather the data they require for their analysis through literature searches. In most cases, once data are retrieved, the search results are examined by eye to determine if they contain the phylogenetic data of interest. Since TreeBASE does not provide a complete phylogenetic data resource, literature searches still have to be performed to ensure thoroughness. Unlike the major sequence databases, phylogenetic tree data does not have to be deposited in a database before it can be published. Currently, the deposition of data in TreeBASE has been voluntary. Also, TreeBASE is not exploited fully because data are difficult to retrieve using search terms that are intuitive to users. Although TreeBASE provides a taxon name search, the returned data are often incomplete. Our hypothesis was that an integrated taxonomic data source could alleviate the problems of using taxonomic names to retrieve data from TreeBASE. Using taxon queries performed on , we show a significant improvement in data retrieval when the same queries were expanded using TCl-Db tables linked to TreeBASE. The following sections describe the taxonomic requirements of TreeBASE, and follow on with a description of TCl-Db, the data warehouse that was developed to meet these needs.

### Taxonomic Requirements of TreeBASE

TreeBASE [8] is a phylogenetic and evolutionary information store containing phylogenies for more than 100,000 taxa. Despite the intrinsic taxonomic content, at design, the developers of TreeBASE purposely excluded taxonomy [8]. The TreeBASE interface  supports six query types: author, citation, study accession number, matrix accession number, taxon and structure. The taxon search, however, does not perform adequately, as it does not effectively support higher taxa queries or synonym and vernacular queries.

From a biologist's perspective, the taxon search option does not return the expected results. The query term 'Aves' currently returns 5 studies (S281, S880, S296, S1166, S433). On closer inspection, there are many more studies containing Aves (birds) within TreeBASE, for example the search term *Gallus *returns a further 2 studies (S1522, S606) and *Diomedea *returns 1 more study (S351). Similarly, the search term *Puffinus *returns no studies, however, using the search terms *Puffinus tenuirostris *or *Puffinus gravis*, the study S714 in which they are located is returned. The species *Puffinus gravis *is also contained in the study S351, however, a search using the taxon name is not successful because the node in the tree is labelled 'Puffinus gravis U74354'. These examples show that higher taxa terms such as 'Aves' and *Puffinus *are not being expanded to include the scientific names they subsume. Queries performed on TreeBASE return only data where the search term matches *exactly *a term contained in the study. As such, the term 'birds', which is the vernacular associated with Aves, returns no data because it is not contained in any study. Similarly, the name *Phoebastria albatrus*, does not return the study S714 in which the currently accepted valid name *Diomedea albatrus*, exists. The taxonomic content and structure of TreeBASE does not support these queries, as query terms are not expanded to include associated terms and, as a result, only partial results are returned. The current data retrieval options within TreeBASE pose a problem for the research community who commonly use taxonomic names as search terms. The research hypothesis studied in this paper is that data retrieval from TreeBASE can be improved by the inclusion of a taxonomic and linguistic infrastructure (a dictionary of synonyms and vernaculars).

The taxonomic requirements that TreeBASE should support are: 1) search terms should expand to include subordinate terms in the classification if they are higher taxa, 2) vernacular queries should be supported and expand appropriately to include the data linked to the scientific names, and 3) any given query should also expand to include data associated with synonyms and out of date usage of a taxon name. These queries are currently not supported by TreeBASE. The developers of TreeBASE purposely excluded taxonomy [8] because there were too many difficulties for a small development team to overcome. The inclusion of a taxonomic infrastructure still poses several challenges. The distributed nature of taxon names and the many data sources in which these are held is a significant problem, as few sources cover the breadth of taxonomic coverage required by TreeBASE. Also, each taxonomic data source uses a particular classification scheme supporting specific taxonomic opinions. Not only do data sources differ in the content they deliver but, even those with similar content may follow different taxonomic opinions and therefore deliver very different classification schemes.

These challenges may be addressed by combining the content of multiple taxonomic data sources and integrating the data into a form that will enable the taxon query extensions we postulate. TCl-Db, a Taxonomy and Classification Database, was developed to increase the accessibility and transparency of taxonomic data by integrating data from the available data sources. It was designed to provide a taxonomic infrastructure to TreeBASE and supports the queries systematists wish to perform.

## Construction and content

### TCl-Db, a Taxonomy and Classification Database

TCl-Db provides a merged view of taxonomic data through a single point of access. The database integrates taxonomic data from several distributed data sources. Architecturally, it forms a warehouse in which taxonomic names from the prominent taxonomic data sources ITIS [22], NCBI [37] and Sp2000 [38] are replicated and maintained in a common structure. These were selected as data sources because of their data content and the ease of downloading and replicating the data structure. Several Aves Checklists [39-43] were made available to us from the early bird project [44], these were initially added in order to evaluate the potential of TCl-Db for data cleaning. Additional checklists data that were requested were Mammal Species of the World [31] and the taxonomic data from GRIN, Germplasm Resources Information Network [45]. A full list of contributing data sources is given in Table 1.

**Table 1:** Summary of data sources.

Data Source	Download Date/Version	Data Source Content
ITIS	January 2004	413,227
ITIS	October 2005	400,863
GRIN	July 2005	94,146
NCBI	September 2004	273,404
NCBI	October 2005	346,840
SP2K	2006 Annual Checklist	1,262,469
ALGAEBASE	SP2K 2005 Annual Checklist	38,150
MSOW	July 2005	6,058

Aves Checklists from early bird project		
nam980612	1998 [44]	12,034
American Ornithological Union	1983 [39]	4,936
American Ornithological Union	1998 [40]	2,755
Sibley and Monroe	1997 [33]	11,932
Peters	1987 [42]	11,267
Clements	2000 [43]	19,305
Bird_names	IOC World bird names 2006	19,313
Morony, Bock, and Farrand	1975 [41]	11,455

Updates have been performed for ITIS and NCBI.

**Figure 1:**
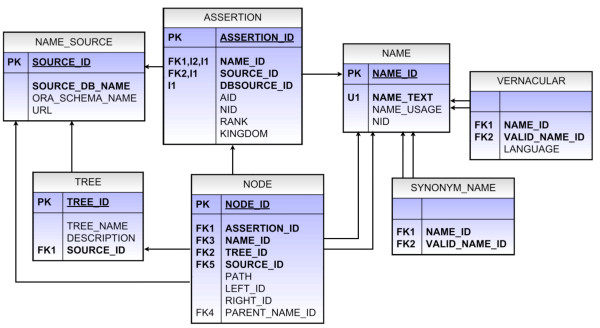
**TCl-Db Database Tables**. TCl-Db tables represent the database implementation. PK means primary key, FK means foreign key, U stands for a uniqueness constraint, and I indicates an integrity constraint (in the table ASSERTION there is a check constraint on the column dbsource_id). In database terminology tables are called relations and columns are called attributes, while the other concepts express integrity constraints which guarantee data quality. Here, we use the terms tables and columns when we refer to the physical model which additionally includes a number of materialised views and database functions and procedures. Those are used during database updates, to keep track of unique identifiers and to maintain referential integrity.

TCl-Db was designed at the early stages of this project, between 2003 and 2004. A full description of the database design and implementation, and an Entity Relationship Diagram (ERD) can be found at . The design phase of TCl-Db identified the entities that support the requirements presented at the start of this section. The entities are as follows. A NAME represents a taxon name. SYNONYMNAME is a taxon name that, although once used as a valid name, was replaced with a new valid name. VERNACULARNAME represents a name used in common language to represent an organism. NAMESOURCE represents the data source from which each NAME entity originated. TREE represents a classification that can be built based on data from a NAMESOURCE and NODES represent the structure of the TREE. The physical database design, implemented using the Oracle database management system [46], is shown in Figure 1. The many to many relationship between NAME and NAMESOURCE is resolved with an association entity, ASSERTION. As well as ensuring the taxonomic names in TCl-Db are tightly bound to their data sources, the ASSERTION entity also increases transparency, by making conflicts and differences between data sources more obvious. This is useful when comparing the composition and data quality of data sources.

The design ensures that each taxon name entering the warehouse is tightly linked to its data source and data source classification. This supplements the concept of data provenance [47,48] and is achieved through the attribute dbsource_id. The dbsource_ids are the database identifiers used at the database source, for example the ITIS dbsource_id for Aves is 174371. These identifiers were stored so that they could be used to link back to the original data source.

### Hierarchical Query Support

To support hierarchical queries on TreeBASE, TCl-Db stores *multiple classifications *giving users the option to choose which hierarchy to traverse in a query. An example of a hierarchical query is the family name *Crocodylidae*. In a hierarchical query this search term would include all the subordinate terms within this family name, i.e., the genera and species names.

**Figure 2:**
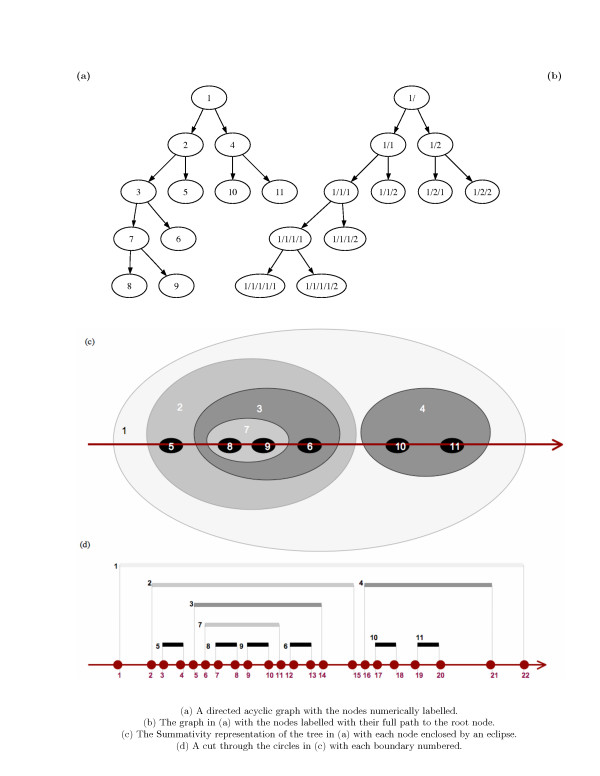
**Nested set and Path representation of a tree**. The directed acyclic graph given in (a) is represented as Materialised paths in (b). The nested sets are shown in (c) using a summativity representation instead of the traditional Tree representation. This representation gives a clearer view of the containment property of hierarchies.

TCl-Db supports three forms of hierarchical queries: Nested sets [49], Materialized Paths [50] and Oracle's 'Connect By' [51]. The calculation of the Nested set and Materialized Path data is depicted in Figure 2. Figure 2a is an example hierarchy with the nodes numerically labelled. The same tree is depicted in Figure 2b, with the nodes labelled with their materialised path and Figure 2c is the summativity representation. Nested sets (Figure 2c) represent a tree using two numbers a left_id and a right_id, the columns left_id and right_id in table NODE (see Figure 1). These left_id and right_ids (nested sets) are calculated using the summativity representation given in Figure 2c. For example, Nodes 10 and 11 are contained within Node 4 which is contained within Node 1. The nested sets reflect this containment, Node 1 having the largest (most inclusive) set of 1, 22. Node 4 has the set (16, 21) which, includes its children Nodes 10 (17,18) and 11 (19,20). The hierarchical query to select all children of node 4 is a simple numerical calculation, see Additional file 1 (Query 1) for an example SQL query using the nested set left_id and right_id. This query uses the function GET_NAME_TEXT which for a given taxon, returns the children of that taxon within the specified hierarchy.

The materialized paths are calculated through a tree walk where a count is incremented as a node is encountered within each level and a new count is created when moving down a level. For example, the root of the tree, the uppermost level, has the path **1**/ and the level below inherits this root path and an additional count reflecting their position below the root. For the two nodes below the root, the path **1/1 **is given to Node 2 and **1/2 **to Node 4. Nodes 10 and 11 are a level below Node 4 and gain their parent path **1/2 **and a new count indicating their location within their parent path thus giving them the paths **1/2/1 **and **/1/2/2**, and so on. Materialised paths are stored in the NODE table in the column path as shown in Figure 1, (see Additional file 1, Query 2, for an example SQL query using materialised paths). The SQL query uses the property that each node inherits its parent path, therefore all children of a node can be selected based on its path being a prefix of the path of its parent. This query uses an additional function GET_ID which returns name_id for a given name, simplifying the query so that it does not require any table joins.

Finally, columns name_id and parent_name_id in the table NODE are used by the 'Connect By' clause (see Additional file 1, Query 3). This method uses the hierarchical relationship modelled as a self-referencing relation. This is the simplest method of modelling the hierarchical relationship between nodes, however, the 'Connect By' clause is specific to Oracle. The addition of the nested sets and materialized paths makes the database portable to other database management systems such as MySQL or PostgreSQL.

### Vernacular Queries and Query Expansion Techniques

Within TCl-Db synonym names are linked to valid names via the table SYNONYM_NAME and vernaculars are linked to valid names through the table VERNACULARS. This supports query expansion of synonyms and vernaculars to Latin names. An example query for the term 'crocodiles' is shown in Additional file 1 as Query 4.

## Utility

TCl-Db was used to test the following hypothesis: *Data retrieval using taxonomic search terms in TreeBASE can be significantly improved by using a data warehouse of integrated taxonomic names and their classifications*.

### Data Sets

The data sets used in this study are summarised in Table 2. The upper section of Table 2 refers to data in the databases TCl-Db and TreeBASE. The lower section of Table 2 refers to data from the TreeBASE query log and the AOL query log. We see within this table that, 29,035 TreeBASE taxa (within the local version of TreeBASE database) were mapped to TCl-Db taxa, and the number of taxon queries from the TreeBASE query log that mapped to the data within TCl-Db were 27,239.

**Table 2:** Summary of data sets used.

Summary of Data Sets						
Database	Taxa	Mapped to TCl-Db	Valid names	Vernaculars	Synonyms	Query Date
						
TCl-Db	1,434,846	1,434,846	916,402	213,602	304,842	01/2006
TreeBASE	56,712	29,035	27,638	540	856	04/2006

Query Log	Queries	Mapped to TCl-Db	Valid names	Vernaculars	Synonyms	Download Date
						
TreeBASE	62,126	27,239	17,006	4624	1010	05/2006
AOL	9,941,434	8,281	3,076	3,590	307	10/2006

The upper section summarises the taxonomic data content for the TCl-Db data warehouse and the taxonomic data content of our local copy of TreeBASE. The lower section summarises the taxonomic data content for the TreeBASE query log and the AOL query log.

### Data Retrieval from TreeBASE

#### TreeBASE Taxon Search Log

The TreeBASE web interface, available at the URL , allows users to conduct taxon queries, queries by a specific matrix identifier, study or tree identifier. These queries return the phylogenetic studies that contain the term that was used in the search. In this study the database structure of TreeBASE was replicated locally so that SQL queries could use the tables within both TreeBASE and TCl-Db.

The taxon queries on TreeBASE came from a script given to us by the TreeBASE developers. The script returned all queries performed using the taxon field in the TreeBASE user interface. These queries and the number of times these queries had been performed were loaded into a database table and given unique identifiers. The data were initially trimmed to remove trailing spaces. Duplicates were removed and so were other non taxon searches, such as queries based on TreeBASE identifiers. There were also several searches for study authors which were removed by comparing the queries to the author names stored in TreeBASE. GenBank Accession number queries were also removed from the data set. The remaining 62,126 queries were then mapped to TCl-Db giving 27,239 distinct taxon queries. Using these 27,239 queries, we compare the data returned in response to the queries directly against a local copy of TreeBASE, downloaded in 2006, and through the wrapper software which uses both TreeBase and TCl-Db. The number of queries that do not return any TreeBASE data is significantly higher than the number of queries that do (16,018 against 11,221). Approximately 50% of the queries posed on TreeBASE were higher taxa queries (of rank genus and above) while 28% were species queries. Of the valid name queries posed against TreeBASE, 71% do not return data, with 94% of the vernacular and 85% of the synonym queries also returning no data. This analysis of the query logs shows that users have been experiencing very poor data retrieval.

#### TCl-Db hierarchical query expansion improves data retrieval

Tables 3 and 4 compare the query effectiveness of TreeBASE alone and TreeBASE terms expanded with taxonomy data from TCl-Db with regard to genus queries in Table 3 and higher taxa in Table 4. Overall, for 6,622 genera queries that return *no data *in TreeBASE, hierarchical query expansion via TCl-Db produces 1,127 trees. The most significant improvement in the number of trees found is seen for 'pinus' (Table 3, from 7 in TreeBASE alone to 123 trees after TCL-DB query expansion) and 'Metazoa' (Table 4 from 5 without TCl-Db to 1,014 additional trees while using NCBI taxonomy within TCl-Db).

**Table 3:** Genus Queries.

Genus	SPECIES count in ITIS	SPECIES count in NCBI	SPECIES count in Sp2000	TREES Returned from genus search on TreeBASE	TREES Returned from species search using TCl-Db
*Platanus*	6	5	6	23	2
***Drosophila***	**378**	**43**	**2,066**	**28**	**88**
*Saccharomyces*	13	62	6	26	73
*Homo*	1	1	1	1	52
*Quercus*	214	89	211	1	5
***Pinus***	**62**	**66**	**57**	**7**	**123**
*Arabidopsis*	2	10	2	9	37
*Acer*	21	79	21	7	9
*Canis*	7	10	7	9	29
*Pan*	2	2	2	1	4
*Escherichia*	21	1	7	0	8
*Acacia*	62	160	1,315	0	4
*Acorus*	2	4	2	13	1
*Phytophthora*	1	74	58	13	29
*Mus*	38	25	38	28	30
*Bacillus*	1	1,450	150	1	5
*Magnolia*	12	76	134	8	4
*Aspergillus*	0	155	185	5	43
*Fusarium*	0	183	85	2	19
*Tetragnatha*	0	21	323	6	6

The number of species within each genus for ITIS, NCBI and Sp2000. Each source shows varied species content for each genus, most notably for *Pinus *and *Drosophila*. The last two columns are: the number of trees returned for the genus queries performed directly on TreeBASE; and the number of additional trees returned using species names found using all three classifications in a hierarchical query in TCl-Db.

**Table 4:** Higher Taxa Queries.

QUERY	Trees Returned using TreeBASE	Trees Returned using TCl-Db with Sp2000 Hierarchy	Trees Returned using TCl-Db with ITIS Hierarchy	Trees Returned using TCl-Db with NCBI Hierarchy
Diptera	7	X	111	106
Lepidoptera	5	41	39	71
Carnivora	12	49	49	65
Animalia	1	954	856	0
Solanaceae	9	80	80	80
Rosaceae	1	42	42	38
Felidae	7	10	10	15
Vertebrata	3	0	408	443
**Fungi**	**8**	**807**	**389**	**814**
Crustacea	2	0	47	38
Chordata	1	433	411	446
**Metazoa**	**5**	**0**	**0**	**1,014**
Poaceae	11	100	100	95
Rodentia	9	100	100	102
Chlorophyceae	6	50	66	50
Cnidaria	3	75	78	79
Arthropoda	5	404	284	371
Primates	7	61	61	61
Aves	8	91	91	87
Reptilia	1	74	74	0
Coleoptera	3	67	45	49
Cetacea	16	47	17	47
Bacteria	2	55	13	35
Ascomycota	9	549	273	540
**Archaea**	**4**	**X**	**0**	**15**
Mollusca	14	75	86	93
Mammalia	12	224	212	221
Fabaceae	11	151	143	151
Asteraceae	11	127	127	156
Insecta	2	325	238	301

Expanding query terms hierarchically increases the number of trees returned from TreeBASE. The first column shows the count of trees found in TreeBASE. The remaining columns show the number of trees returned using hierarchical query expansion on TreeBASE using Sp2000, ITIS and NCBI classifications. The table highlights the importance of including more than one hierarchy. For instance, the query 'Metazoa' returns no data when using the ITIS or Sp2000, and 1014 when using NCBI. Also, for 'Fungi' we see that NCBI and ITIS differ. In some cases the hierarchical query failed, denoted with an X. For example, as the term 'Archaea' is both a genus and superkingdom in SP2K, the hierarchical query fails.

#### TCl-Db synonym and vernacular query expansion has a positive impact on data retrieval quality

Vernacular queries on TreeBASE perform particularly poorly (Table 5), as most commonly submitted queries return no results, with the exception of query 'primates' which returns two trees. While vernaculars are not the most frequently used search terms, TCl-Db allows these terms to expand to Latin names. For example, 'acacia' (Latin *Robinia pseudoacacia*) returns no data in TreeBASE, while the Latin term, related to acacia, returns 2 trees, and 'yeast' (Latin *Saccharomyces cerevisiae*) has no direct hits in TreeBASE, but returns 70 trees when TCl-Db is used (a similar observation was made by Jensen et al in [6]). In TCl-Db, the inclusion of the alternative Latin names significantly improves the quality of data retrieval. For those queries that translate to higher taxa names, data retrieval can be further enhanced by performing a hierarchical query.

**Table 5:** Vernacular Query terms.

Query	TCl-Db Query	TreeBASE alone	TCl-Db with TreeBase
maple	Acer	0	7
primates	primata	2	3
pine	*Pinus brutia*	0	2
pine	Pinus	0	7
eubacteria	Bacteria	0	2
mouse	*Mus musculus*	0	28
birds	Aves	0	8
dog	*Canis familiaris*	0	19
mammals	Mammalia	0	12
human	*Homo sapiens*	0	52
elm	Ulmus	0	2
**Acacia**	*Parkinsonia aculeata*	**0**	**2**
**Acacia**	*Acacia ampliceps*	**0**	**2**
**Acacia**	*Robinia pseudoacacia*	**0**	**10**
yeast	*Saccharomyces cerevisiae*	0	70

The most common vernacular queries with Latin names and the number of trees found for each query.

Expanding search terms with synonyms also improves data retrieval. There were 868 synonym queries that returned no data using TreeBASE. In response to these queries, TCl-Db returned 594 trees by expanding the search term with valid names linked to synonyms.

An alternative query log from AOL [52] was analysed for taxon searches. Taxon searches were extracted from this log for the purposes of providing a test set of queries that can be used to test our TCl-Db TreeBASE wrapper. Surprisingly, from the AOL data we see that vernacular queries were only marginally more frequent than scientific name queries (see Table 2) i.e. 3590 against 3076 out of the 8281 AOL taxon queries.

#### TCl-Db Provides Taxonomic Awareness for TreeBASE

The lack of taxonomic content in TreeBASE is responsible for poor data retrieval. Previous studies have also highlighted this. The taxon names in a 2004 snapshot of TreeBASE were mapped previously to the databases IPNI, ITIS, NCBI, and uBio in TBmap [53], and this work comments on the importance of internal consistency within a database system and the requirement for data validation. TCl-Db can also be used for this purpose and part of that analysis was replicated here in an *automated way*. Through SQL queries we mapped 28,876 TreeBASE taxa to taxa in TCl-Db. The distribution of TreeBASE names, grouped by taxonomic rank, is shown in Table 6. This shows that the majority of TreeBASE names are species, while the majority of queries performed on TreeBASE are higher taxa. It is not surprising, therefore, that data retrieval is poor. The lack of taxonomic support in TreeBASE means that queries do not return data because the query terms are not understood by the system. One way to improve this, as shown above, is to increase 'the vocabulary' of the database. The superimposition of a taxonomy onto the TreeBASE structure makes sure the queries are understood by the system and makes it significantly more user friendly.

**Table 6:** Proportion of Higher Taxa Queries within TreeBASE Query log.

	TreeBASE database	TreeBASE Query Log
Subspecies	218	145
Species	23,105	7,781
Higher Taxa	5,086	13,558

TreeBASE taxon content within the TreeBASE taxon query log. The difference between the distribution of taxon names in TreeBASE and the TreeBASE query log is large. The vast majority of taxa in TreeBASE are species (left) while the types of queries performed on TreeBASE concern higher taxa.

Although a number of integrated database systems already exist and store names from multiple sources, the classifications of those names are not stored and a user cannot freely choose the classification that suits their work best. TCl-Db was developed because Sp2000 and uBio could not meet the requirements we gathered. The specific shortcoming of Sp2000 was that it did not support multiple classifications, while uBio could not effectively link to TreeBASE. However, uBio extended its services to include classifications [54] which is accessible only through a web service.

TCl-Db supports a number of *novel functions *not included within other systems. First, it performs hierarchical searches through a choice of three classifications. Providing a higher taxon name as a query returns names contained within the hierarchy. Second, it expands terms (with synonyms and vernaculars) to include valid names that are associated with them. These queries are similar to 'drill down' browsing searches and 'fuzzy' queries using generalised terms. These queries are supported by a local copy of TreeBASE accessed through a web based wrapper.

**Figure 3:**
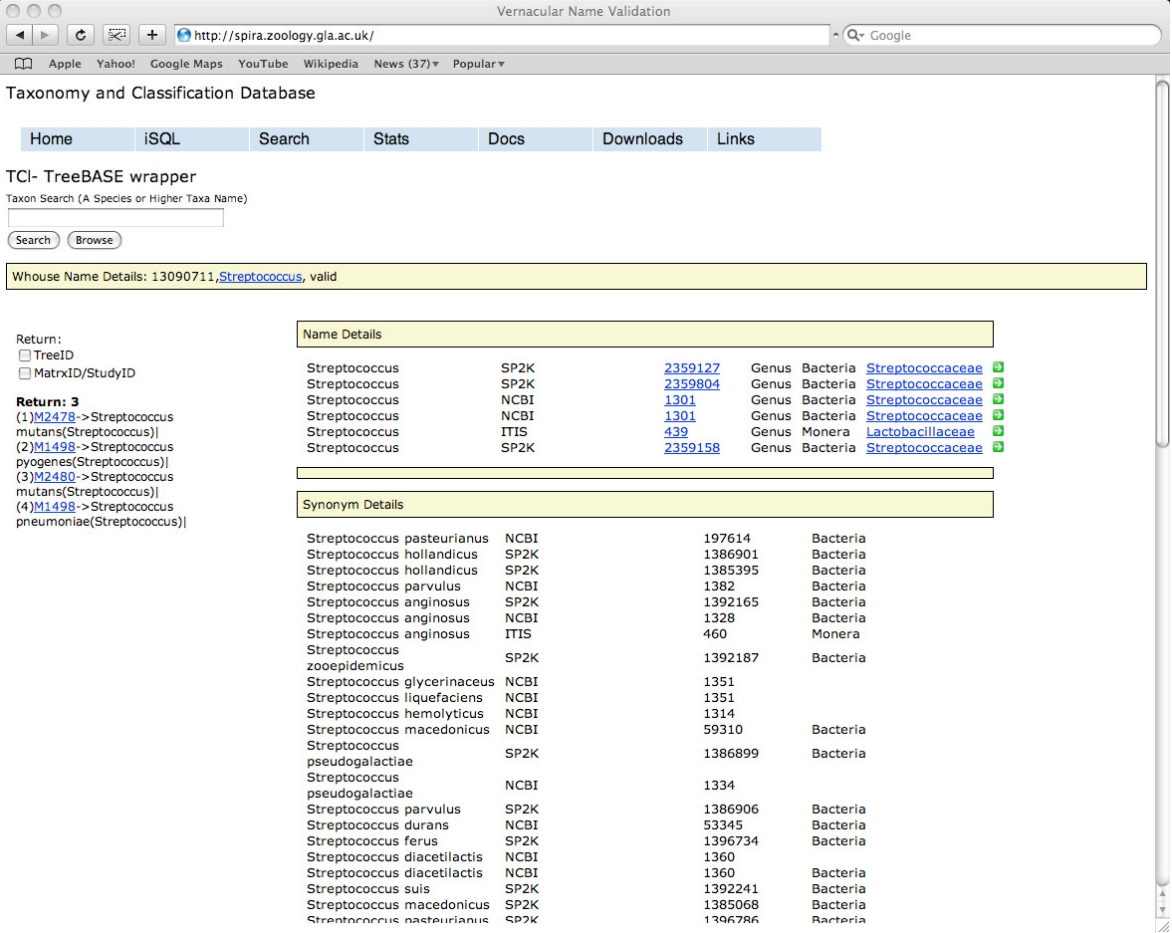
**TreeBase Wrapper – Search Page**. This page can be accessed from the URL . In response to the query 'Streptococcus', TCl-Db wrapper returns three distinct taxa present in four trees (left pane). The right pane shown shows taxa details for 'Streptococcus' from the data sources included in TCl-Db.

**Figure 4:**
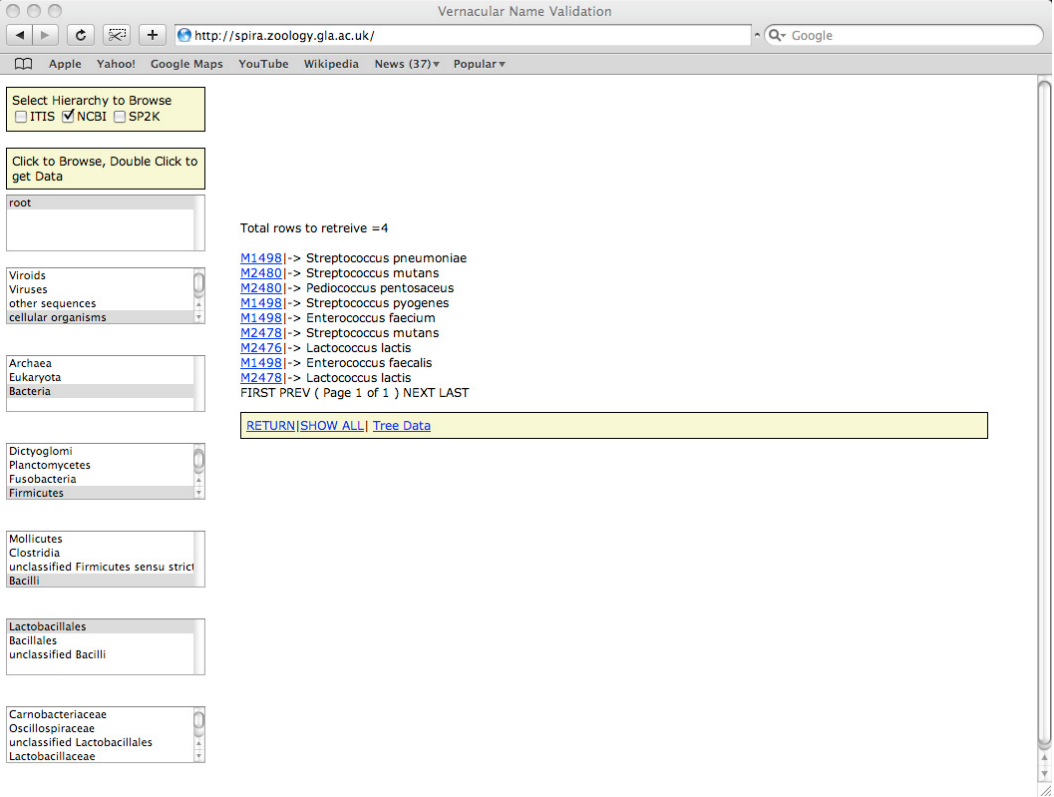
**TreeBase Wrapper – Browse Page**. This page can be accessed from the URL . The NCBI hierarchy is traversed to 'Lactobacillales', which returns 4 distinct trees (M1498, M2480, M2478 and M2476). The query is started by selecting the classification using the select boxes in the top left, the choices are ITIS, NCBI and SP2K. The hierarchy is traversed with a single mouse click through each level as it appears. A double click on a taxon name triggers a TCl-Db query through TreeBASE.

The interface to TCl-Db provides both a search form (Figure 3) and a classification browse page (Figure 4) which returns either TreeBASE *treeids *or *studyids *which link to the current online TreeBASE interface via hyper links. The web interface enables the user to enter vernacular names as search terms. These searches return a list of linked taxon names from which the user can select. For example, entering the search term 'birds' will return a link to the term 'Aves'. The search form also enables the user to use an approximate spelling, as in Google's 'did you mean' link. For example, the search term 'Caenorabditis' returns no data but suggests 'Caenorhabditis' as an alternative. Hierarchical queries are also supported. Once a search term is entered, the system returns a list of classifications. Once a classification is selected, the query expands to subordinate terms within the classification and each term is searched through TreeBASE. Additionally, a browse function is supported. It allows the users to first select which hierarchy they wish to browse (ITIS, NCBI or Sp2000), and then select the taxon for which they want to retrieve data.

## Discussion

The version of TreeBASE on which this analysis was based is to be replaced by CIPRES as TreeBASE2 [23]. Although a prototype was due for release in July 2006, it is not available yet. The new improved TreeBASE schema has a Taxon module which looks to rectify many of the data retrieval issues currently experienced by users. It is difficult to see from the available documentation and schema exactly how hierarchical and vernacular queries will be supported in TreeBASE2, and until the system comes online, our web application makes clear the advantages of supporting taxon queries, and the benefits of query expansion.

Phylofinder [9] also shows how data retrieval can be improved with the inclusion of a taxonomy. It uses the NCBI classification and makes use of TBmap [53] to deal with taxa names that are not included in NCBI. On the whole, Phylofinder does improve data retrieval, however, the inclusion of just one classification limits the higher taxon queries that can be performed to only those included in NCBI and TBmap. Table 7 shows a selection of higher taxa terms from the ITIS classification, and shows that data retrieval in Phylofinder is still limited, as for instance the query 'Craspedomonadales' returns no hits in Phylofinder and 35 when TCl-Db is used, and 'Pinales', with no hits in Phylofinder, brings 37 trees when routed via TCl-Db. This is partly due to the fact that TBmap has a restricted scope, as not only is the mapping based on a 2004 snapshot of TreeBASE, but also the mappings are limited to the taxa contained in TreeBASE. As a result, many higher taxa queries are not well supported. Although TCl-Db, uses a 2006 snapshot of TreeBASE it is only marginally outperformed by Phylofinder which uses a more recent version of TreeBASE. The queries 'Aves' and 'Puffinus', exemplified originally, return 1 more tree and 6 more trees respectively in Phylofinder. The inclusion of more than one classification scheme and the support for vernacular queries make the approach used by TCl-Db superior to that used by Phylofinder. Phylofinder is based on mappings that are already out of date, therefore, its shelf life is limited, whereas TCl-Db performs mappings to TreeBASE automatically, and, therefore, will be able to provide a more useful resource in the long term.

**Table 7:** ITIS higher taxa queries in Phylofinder and TCl-Db.

Query	Phylofinder (trees found)	TCl-Db (trees found)
Aristolochiales	0	7
Bromeliales	1	16
Calycerales	0	4
Schistostegiales	0	1
Aulacoseirales	0	1
Centrales	0	5
Chromalinales	0	4
**Craspedomonadales**	**0**	**35**
Leitneriales	0	2
Lithodesmiales	0	2
Plumbaginales	0	10
Polygalales	1	14
Hydrocharitales	0	3
**Pinales**	**0**	**37**
Eriocaulales	0	12
Fissidentales	0	6
Papaverales	0	8
Cryptonemiales	0	2
Biddulphiales	1	3
Restionales	0	8

A sample of ITIS higher taxa in the Plant Kingdom with the number of trees returned in Phylofinder and TCl-Db. Terms Pinales and Craspedomonadales, in bold, return large numbers of hits in TCl-Db.

## Future work

### Data Freshness

One of the challenges is data maintenance within TCl-Db. Even though the system was developed with TreeBASE as the primary source of phylogeny data, there may be other database systems that could benefit from the inclusion of a taxonomy. We need to keep data sets current for the system to be useful in the long term and to other consumers. Updates to NCBI and ITIS classifications have been performed manually and the process has highlighted maintenance issues that need to be addressed to support automated updates which would keep the data current. This is the focus of current work. Currently data updates are performed when requested, we endeavour to update the ITIS and NCBI data at least yearly. The addition of new or updated checklist data can be added on request.

### Semantic Web Technologies

The core of TCl-Db work is data integration. From the database perspective, data warehousing and data integration [55] involve gathering data from several silos and mapping those into a common schema. Integration is achieved by issuing queries on this common structure. On the web, however, data are not integrated physically but are linked using URLs, which provides a certain degree of flexible adjustment, as sources evolve. In the next generation of the web, resources, given correct meta-data [56], could be linked automatically via ontological annotations [57]. Semantically annotated data will have meaning to computers and not just to the users browsing them [58], which enables automatic data matching, integration and translation. Semantic web technologies should be able to support automated linking of phylogenetic and taxonomic resources [59]. Making taxonomic data interoperable [60] would be of great benefit, as it would remove the need for carefully orchestrated updates, which would be replaced by distributed web querying. Also, the distributed nature of systematics lends itself to the semantic web ethos. Potentially, semantic web technologies will reduce the need for data warehousing, and replace the centralised approach to data management with a distributed one [61]. The future development of TCl-Db will make use of semantic technologies for data integration and support greater interoperability of taxonomy and phylogeny systems.

## Conclusion

The lack of taxonomic intelligence in TreeBASE makes data retrieval ineffective in some cases. Our hypothesis that data retrieval can be improved through the inclusion of taxonomic meta-data is well substantiated. We clearly show that where TreeBASE finds little data, TCl-Db delivers improved results. TCl-Db provides an infrastructure supporting effective data retrieval within TreeBASE by using taxon names as search terms. The analysis we presented shows the importance of this meta-data in supporting queries found in query logs. Additionally, via the inclusion of vernaculars and synonyms, additional data can be found in TreeBASE. The use of an amalgamated taxonomy data warehouse also addressed the issues of taxonomic coverage and the differing opinions in taxonomy, and supports the comparison of taxonomy and data coverage in several contexts.

## Availability and requirements

The wrapper which expands queries with information from TCl-Db can be accessed at the URL  and has been tested on Mozilla Firefox version 2 and Safari version 3. Database dumps for Oracle and MySQL can be found at .

## Abbreviations

TCl-Db: Taxonomy and Classification Database; ITIS: Integrated Taxonomic Information System; Sp2000: Species 2000; NCBI: National Center for Biotechnology Information; uBio: Universal Biological Indexer and Organiser; AOL: America OnLine; CIPRES: CyberInfrastructure for Phylogenetic RESearch.

## Authors' contributions

NA was the primary designer and developer of TCl-Db, and wrote the paper. EH provided some suggestions, and edited parts of the manuscript. Both authors read and approved the final version.

## Supplementary Material

Additional file 1 **SQL Queries**. The data provided represent example SQL queries for each of the hierarchical queries (Queries 1 – 3) and for expanding vernaculars to valid names (Query 4). Click here for file
