# Supplementary material for: Improved data retrieval from TreeBASE via taxonomic and linguistic data enrichment
Source: BMC Evol Biol. 2009 May 8;9:93. doi: 10.1186/1471-2148-9-93 (PMC2685121; doi:10.1186/1471-2148-9-93)
Supplement: Additional file 1 — SQL Queries. The data provided represent example SQL queries for each of the hierarchical queries (Queries 1 – 3) and for expanding vernaculars to valid names (Query 4). [file 1471-2148-9-93-S1.pdf]

Query1

```
FUNCTION UseNestedSets (
name VARCHAR, tree NUMBER)
– input taxon name
– input source tree
RETURN VARCHAR
IS
– output taxon names
outName VARCHAR(4000) := NULL;

BEGIN
– hierarchical query using nestedSet left_Id and right_Id
SELECT get_name_text (node.name_Id)
INTO UseNestedSets.outName
FROM node
WHERE tree_id = tree
AND node.left_id BETWEEN
(SELECT left_id FROM node
WHERE name_id = get_id(UseNestedSets.inName)
AND tree_id = UseNestedSets.inTree)
AND
(SELECT right_id FROM node
WHERE name_id = get_id(UseNestedSets.inName)
AND tree_Id = UseNestedSets.inTree);

RETURN outName;
END;
```

Query2

```
FUNCTION UsePaths (
name VARCHAR, tree NUMBER)
– input taxon name
– input source tree
RETURN VARCHAR
IS
– output taxon names
outName VARCHAR(4000) := NULL;
– output path string
outPath VARCHAR(4000) := NULL;

BEGIN
– SQL statement for path string
SELECT path INTO UsePaths.outPath
FROM node
WHERE name_Id = get_id(name);

– hierarchical query using path string
SELECT get_name_text(node.name_id)
INTO UsePaths.outName
FROM node
WHERE path LIKE UsePaths.outPath%
AND tree_Id = UsePaths.input tree;

RETURN outName;
END;
```

Query3

```
FUNCTION UseConnectBy (
– input taxon name
inName VARCHAR,
– input source tree
inTree NUMBER)
– outputs taxon names
RETURN VARCHAR
IS
outName VARCHAR(4000) := NULL;

BEGIN
– hierarchical query using connect by
SELECT get_name_text (node.name_id)
INTO UseConnectBy.outName
FROM node
WHERE tree_id = inTree
START WITH (get_id(UseConnectBy.inName)
AND tree_id = UseConnectBy.inTree)
CONNECT BY PRIOR parent_name_id = name_Id;
RETURN outName;
END;
```

Query4

```
FUNCTION Vernacular2Valid (
– input vernacular name
inName VARCHAR)

– outputs taxon names
RETURN VARCHAR
IS
outName VARCHAR(4000) := NULL;

BEGIN
– vernacular query
SELECT get_name_text(valid_name_id)
INTO Vernacular2Valid.outName
FROM vernacular
WHERE name_id IN (

SELECT name_id
FROM name
WHERE name_text = Vernacular2Valid.inName );
RETURN outName;
END;
```
